# Using a web-based game to prevent posttraumatic stress in children following medical events: design of a randomized controlled trial

**DOI:** 10.3402/ejpt.v4i0.21311

**Published:** 2013-07-26

**Authors:** Meghan L. Marsac, Kristen L. Kohser, Flaura K. Winston, Justin Kenardy, Sonja March, Nancy Kassam-Adams

**Affiliations:** 1 Child and Adolescent Psychiatry and Behavioral Science, The Children's Hospital of Philadelphia, Philadelphia, PA, USA; 2 Department of Psychiatry, University of Pennsylvania, Philadelphia, PA, USA; 3 Department of Pediatrics, The Children's Hospital of Philadelphia, Philadelphia, PA, USA; 4 Division of General Pediatrics and Leonard David Institute for Health Economics, University of Pennsylvania, Philadelphia, PA, USA; 5 Schools of Medicine and Psychology, University of Queensland, Brisbane, Australia; 6 Department of School of Psychology, Counselling and Community, University of Southern Queensland, Toowoomba, Australia; 7 Department of Pediatrics, University of Pennsylvania, Philadelphia, PA, USA

**Keywords:** Trauma, early intervention, prevention, Internet, PTSD

## Abstract

**Background:**

Medical events including acute illness and injury are among the most common potentially traumatic experiences for children. Despite the scope of the problem, only limited resources are available for prevention of posttraumatic stress symptoms (PTSS) after pediatric medical events. Web-based programs provide a low-cost, accessible means to reach a wide range of families and show promise in related areas of child mental health.

**Objectives:**

To describe the design of a randomized controlled trial that will evaluate feasibility and estimate preliminary efficacy of Coping Coach, a web-based preventive intervention to prevent or reduce PTSS after acute pediatric medical events.

**Method:**

Seventy children and their parents will be randomly assigned to either an intervention or a waitlist control condition. Inclusion criteria require that children are aged 8–12 years, have experienced a medical event, have access to Internet and telephone, and have sufficient competency in the English language to complete measures and understand the intervention. Participants will complete baseline measures and will then be randomized to the intervention or waitlist control condition. Children in the intervention condition will complete module 1 (Feelings Identification) in the hospital and will be instructed on how to complete modules 2 (Appraisals) and 3 (Avoidance) online. Follow-up assessments will be conducted via telephone at 6, 12, and 18 weeks after the baseline assessment. Following the 12-week assessment, children in the waitlist control condition will receive instructions for completing the intervention.

**Results:**

Primary study outcomes include data on intervention feasibility and outcomes (child appraisals, coping, PTSS and health-related quality of life).

**Discussion:**

Results will provide data on the feasibility of the implementation of the Coping Coach intervention and study procedures as well as estimations of efficacy to determine sample size for a larger study. Potential strengths and limitations of this design are discussed.

Events related to injury, acute medical illness, and medical treatment are among the most common traumatic experiences of children (Murray & Lopez, 1996). Worldwide, injuries are a leading cause of death and disability for youth (Peden, 2008), with 20 million children suffering unintentional injuries annually in the United States alone (Grossman, 2000). Countless children across the globe also experience illnesses that involve disruptive, painful, and potentially traumatic disease episodes and treatment procedures (Marks & McQueen, 2001). For many children, it is not physical recovery but psychosocial sequelae that determine functioning after acute traumatic events. A meta-analysis of medical traumatic stress studies found that an average of 19% children with injuries and 12% children with illness experience significant posttraumatic stress symptoms (PTSS) following their medical event (Kahana, Feeny, Youngstrom, & Drotar, 2006). PTSS are a key predictor of functional outcome and health-related quality of life (HRQOL), may interfere with adherence to medical regimens, and have been linked to poorer health outcomes (Graham-Bermann & Seng, 2005; Holbrook et al., 2005; Landolt, Buehlmann, Maag, & Schiestl, 2009; Landolt, Vollrath, Gnehm, & Sennhauser, 2009; Zatzick et al., 2008). Thus, PTSS resulting from medical events are a major health concern for children.

While research has suggested risk and protective factors and mechanisms involved in the development of psychological symptoms following medical trauma, this knowledge has not been translated into widely available preventive interventions (Sabin, Zatzick, Jurkovich, & Rivara, 2006; Ziegler, Greenwald, DeGuzman, & Simon, 2005). The Internet provides a low-cost, accessible method for delivery of psychological and health information and interventions. More than 75% of US and European children have Internet access at home (Child Trends DataBank, 2003; Livingstone & Haddon, 2009). Furthermore, there is growing empirical support for the efficacy of using the Internet to deliver cognitive behavior interventions to children and parents (Magee, Ritterband, Thorndike, Cox, & Borowitz, 2009; March, Spence, & Donovan, 2009; Ruzek et al., 2011; Spence et al., 2011; Spence, Holmes, March, & Lipp, 2006). In particular, Internet interventions have demonstrated efficacy in delivering education and intervention to large numbers of individuals exposed to traumatic events (e.g., military service members; Ruzek et al., 2011). Beyond their broad accessibility, Internet-facilitated interventions may also represent a potentially cost-efficient avenue for the delivery of preventive psychosocial care in the acute phase posttrauma (Mouthaan, Sijbrandij, Reitsma, Gersons, & Olff, 2011). Web-based preventive interventions have the potential to provide children and parents with accessible, just-in-time psycho-education and practical tools for coping with the aftermath of a traumatic medical event.

Coping Coach is an innovative and interactive e-health application that aims to prevent persistent traumatic stress and promote emotional recovery in school-age children after an acute traumatic event. Content and interactive activities were developed for the intervention based on evidence regarding the etiology of traumatic stress, risk and protective pathways, and effective interventions for trauma and anxiety in children.

## Targets for preventive intervention

Risk and etiological variables associated with children's psychological reactions after exposure to acute traumatic events include pretrauma factors (pre-existing trauma exposure or psychological symptoms); peri-trauma factors (perceived life threat, acute heart rate, and physiological arousal), and posttrauma factors (acute stress reactions, maladaptive cognitive appraisals, types of coping Bryant, Salmon, Sinclair, & Davidson, 2007a, b; Ehlers, Mayou, & Bryant, 2003; Kahana et al., 2006; Kassam-Adams, 2006; Landolt, Vollrath, & Ribi, 2002; Salmon, Sinclair, & Bryant, 2007; Zehnder, Prchal, Vollrath, & Landolt, 2006). While pre-existing factors are not amenable to change, malleable posttrauma etiological factors provide an opportunity for secondary prevention programs to enhance adjustment (i.e., improve HRQOL) and reduce the development or escalation of psychological symptoms (Graham-Bermann & Seng, 2005; Holbrook et al., 2005; Landolt, Vollrath, Gnehm, & Sennhauser, 2009; Zatzick et al., 2008). Potential malleable targets for the prevention of posttraumatic stress in children that are supported by research evidence include negative appraisals about safety and vulnerability to future harm (Bryant et al., 2007a; Ehlers et al., 2003; Meiser-Stedman, Dalgleish, Glucksman, Yule, & Smith, 2009), the coping strategy of seeking social support (Stallard, Velleman, Langsford, & Baldwin, 2001), and early avoidance behaviors (Ebata & Moos, 1991). Given the strong support for the effectiveness of cognitive-behavioral theory (CBT) interventions to treat mood and anxiety symptoms in children and teens (Cohen & Mannarino, 2008; Kenardy, Spence, & Macleod, 2006; March et al., 2009; O'Kearney, Kang, Christensen, & Griffiths, 2009; Spence et al., 2011), secondary prevention may also benefit from a CBT approach. Coping Coach uses CBT principles, integrating interactive activities and content, to address each malleable intervention target (i.e., appraisals, social support, avoidance behaviors) in the early posttrauma period.

## Web-based health interventions

Web-based programs have improved symptom management and adherence to medical regimens for asthma, pain, encopresis, and obesity in children (Stinson, Wilson, Gill, Yamada, & Holt, 2009), as well as symptoms of anxiety and depression in adults, children, and adolescents (Kenardy et al., 2006; O'Kearney et al., 2009). For example, BRAVE-Online, a cognitive-behavioral intervention for children with anxiety disorders, has shown efficacy in reducing anxiety symptoms in up to 75% of youth users at follow-up (March et al., 2009; Spence et al., 2011, Spence et al., 2006). To date, the only web-based preventive intervention that has shown promise in preventing psychological symptoms in children exposed to medical trauma is “Kids and Accidents,” designed by Kenardy and colleagues (Cox & Kenardy, 2010). This intervention combined print information for parents with a basic, informational website (kidsaccident.psy.uq.edu.au) for youth. An initial randomized controlled trial (RCT) (*N=*56) showed not only a reduction of anxiety and a trend for reduced PTS symptoms among higher risk children but also suggested a need for greater engagement of children with site activities (Cox & Kenardy, 2010). Given the success of BRAVE-online with its interactive features, it may be that increasing the interactive nature of Kids and Accidents could improve its effectiveness. Much remains unknown about optimizing content and implementing web-based preventive interventions for youth exposed to acute medical trauma. A thorough evaluation of web-based interventions, such as Coping Coach, can help improve understanding about the most potent intervention targets.

## Current study

We created the Coping Coach intervention to address the unmet need of supporting children emotionally following acute traumatic events. In this paper, we describe the design of a RCT evaluating the impact of the Coping Coach intervention on proximal targets (coping, appraisals) and later child health outcomes (PTSS, HRQOL). Specific objectives for the RCT are three-fold: (1) to determine whether the intervention will be used as intended; (2) to examine the feasibility of study procedures; and (3) to estimate efficacy to determine the sample size needed for an outcome evaluation study. In this paper, we share the design of this RCT, including strengths and limitations, with the intention of informing future study designs for web-based intervention research.

## Coping Coach intervention description

**Fig. 1:**
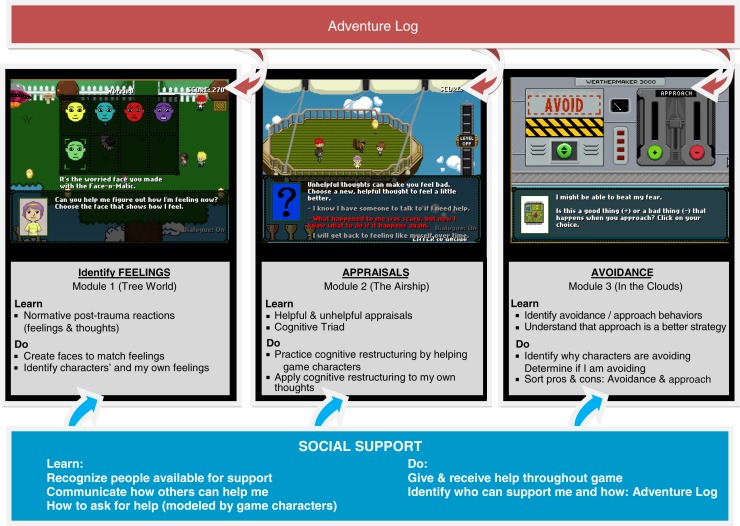
Coping coach intervention overview.

Coping Coach utilizes an interactive, developmentally appropriate, game-like format to provide practical information and teach children adaptive coping strategies. The intervention is developed for the early posttrauma period and will be implemented as a novel, cost-effective, and widely accessible delivery mechanism—the Internet. Children are primary users of the intervention, with parents providing supervision. Coping Coach contains three modules (focusing on feelings identification, appraisals, and avoidance) that are used sequentially, and an adventure log that spans all modules. Identifying and using social support is folded throughout the intervention, as children engage with characters and provide and receive help and support. Each module can be completed in 20–30 min and can be repeated to solidify skills and learning. The feelings module targets recognition and communication of emotions after potentially traumatic experiences. The appraisals module teaches the “cognitive triad” including the relationship of helpful or unhelpful thoughts to feelings and behavior. The avoidance module aims to reduce reliance on avoidance as a coping response. The adventure log encourages children to personalize their learning to their own experience and reinforces the skills in each module. Every module of Coping Coach requires the child to interact with the game content, going beyond information provision, with the aim of children learning through experience. While children are primary users of Coping Coach, parents are encouraged to support their child's engagement in Coping Coach. The website which presents the game for children also provides information for parents about how and when to seek professional help for their child. See Fig. 1 for an overview of the intervention.

## Method

### Participants

Our study sample will consist of 70 children receiving medical treatment for acute medical events and their parents. We define an acute medical event as a sudden, unexpected, and new medical event for a child (i.e., new injury or illness diagnosis, or a sudden exacerbation of a chronic condition). Inclusion criteria for this study are the following: (1) child is aged 8–12 years; (2) child has experienced an acute medical event within the past 2 weeks; (3) child perceives the event as potentially traumatic, based on a brief set of validated screening questions administered prior to enrollment (i.e., meeting DSM-IV A2 criteria; *see Assessments*); (4) child's Glasgow Coma Score is greater than 12; (5) child speaks English well enough to complete measures and understand the intervention; and (6) child has access at home to the Internet and telephone. Exclusion criteria are the following: (1) child's current medical condition or cognitive limitations preclude participating; (2) child's acute medical event is due to family violence or suspected child abuse; (3) child or parent has been arrested or is subject to legal proceedings related to the medical event; and (4) in the index event, child or parent was a perpetrator of violence.

### Study design

Once eligibility is established and consent and assent are obtained, participants will complete baseline measures and then be randomized to either the intervention or waitlist control condition. At the beginning of the study, a random number generator will be used to determine the order of the randomization (intervention condition=35; waitlist control condition=35). Research staff (who will not be enrolling participants) will prepare sealed envelopes, which will only be opened upon the completion of the baseline measures. Those in the intervention condition will complete an initial Coping Coach activity and receive log-in instructions to complete the remaining activities online over the next month. Those in the waitlist control condition will receive log-in instructions for Coping Coach following the 12-week assessment. Follow-up assessments will be conducted with all participants over the phone at 6, 12, and 18 weeks post-baseline. The study protocol has been reviewed and approved by the Institutional Review Board at The Children's Hospital of Philadelphia and is registered at clinicaltrials.gov

### Procedure

Potential participants will be identified via the hospital registries. Children who meet initial eligibility criteria and their caregivers will be approached by a member of the research team who will explain the study and invite participation in screening. After verbal consent and assent are obtained to participate in the screening phase of the study, we will collect basic demographic information and the child will complete four questions (subjective rating of the event as potentially traumatic). If the child's responses indicate that the event is perceived as potentially traumatic, the child is eligible for the RCT portion of the study (see *Assessments*). Those eligible will be offered participation in the full study, will provide written consent and assent, and will complete baseline measures (i.e., demographics, child trauma history, coping and coping assistance, cognitions and appraisals, HRQOL, and PTSS). Subsequently, participants will be randomized to one of two study conditions: 35 to the intervention and 35 to waitlist control.

**Fig. 2:**
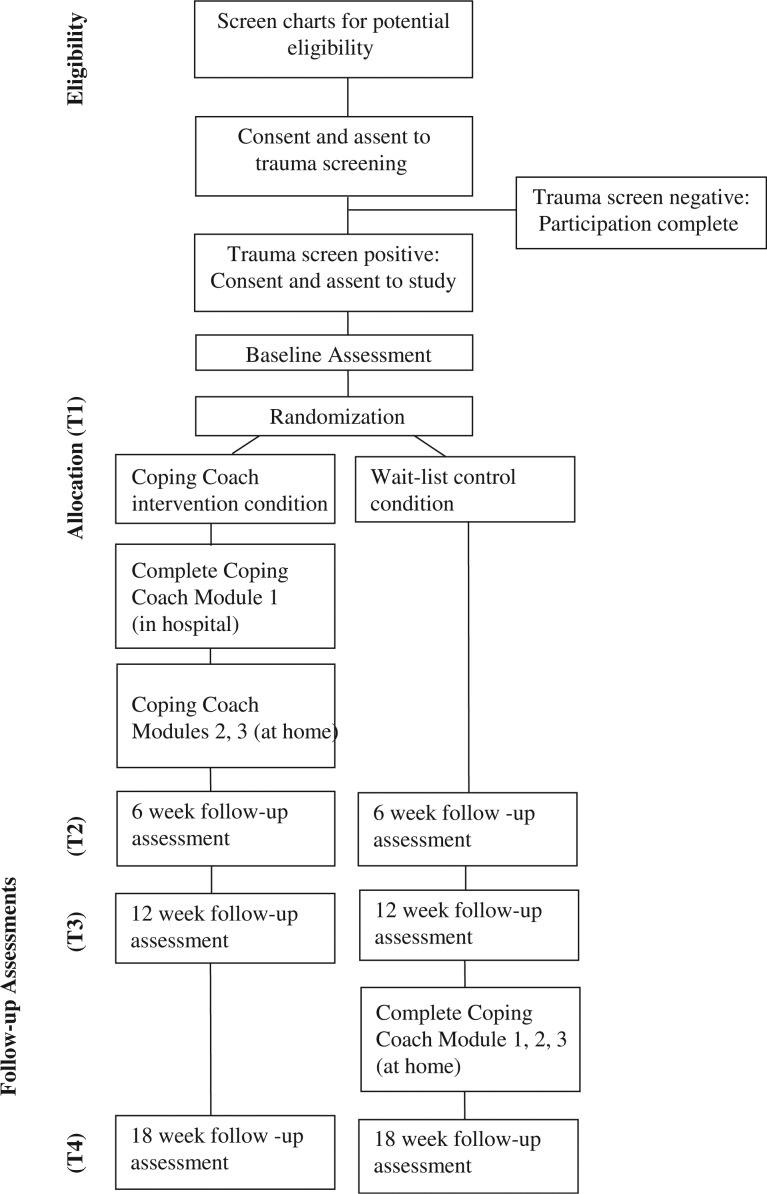
CONSORT diagram displaying study enrollment and randomization.

Those in the intervention condition will complete the first module of Coping Coach (i.e., feelings identification) and will receive log-in instructions to complete the intervention online over the following month. After the 12-week assessment, those in the waitlist control condition will receive log-in instructions. Between baseline and 6 weeks, parents and children in the intervention group will receive tailored weekly reminders via email, text, or phone to encourage children to complete the remaining Coping Coach activities. Between 12 and 18 weeks, participants in the waitlist control condition will receive these reminders. Follow-ups will be conducted with all participants at approximately 6, 12, and 18 weeks post-baseline assessment via the telephone, by research assistants blinded to the child's study condition. Follow-up assessments include measures of coping and coping assistance, cognitions and appraisals, HRQOL, and PTSS. Measures of intervention satisfaction and engagement will be administered separately at either 6 or 18 weeks, depending on the intervention condition. See Fig. 2 for an overview of the study procedures.

### Assessments

See Table 1 for a summary of measures included at each assessment time-point. See below for detailed measure descriptions.

**Table 1:** Construct and measures administered by time point

		Time of assessment			
		
Construct	Measure	T1	T2	T3	T4
Child background characteristics	Demographics questionnaire	X			
Prior trauma exposure	UCLA PTSD Index (Pynoos, Rodriguez, Steinberg, Stuber, & Frederick, 1998)	X			
Coping strategies	HICUPS (Ayers, Sandler, West, & Roosa, 1996)	X	X	X	X
Coping assistance strategies	PSCQ (Miller, Kliewer, Hepworth, & Sandler, 1994)	X	X	X	X
Trauma-related appraisals	CPTCI & AAQ (Ellis, 2008; Meiser-Stedman, Smith, et al., 2009)	X	X	X	X
HRQOL	PedsQL (Varni, Seid, & Rode, 1999)	X	X	X	X
Child PTSS	CPSS (Foa, Johnson, Feeny, & Treadwell, 2001)	X	X	X	X
Parent PTSS	PCL (Weathers & Ford, 1996)	X	X	X	X
Help-seeking	Health & recovery questionnaire (Marsac, Cirilli, Kassam-Adams, & Winston, 2011)			X	
Satisfaction and engagement	Satisfaction & engagement questionnaire		X		X

HICUPS=How I Coped Under Pressure Scale; PSCQ=Parent Socialization of Coping Questionnaire; CPTCI=Child Posttraumatic Cognitions Inventory; AAQ=Adaptive Appraisals Questionnaire; HRQOL=health-related quality of life; PedsQL=Pediatric Quality of Life Inventory; PTSS=posttraumatic stress symptoms; CPSS=Child PTSD Symptom Scale; PCL=PTSD Checklist.

### Eligibility screening

#### Screen of potentially traumatic events

This measure assesses children's subjective rating of the event as potentially traumatic (Kassam-Adams, 2006). Children will provide a brief description of the medical event(s) that brought them to the hospital and will answer a validated four-item screen to assess whether the child perceives the event as potentially traumatic. Each item is rated on a 3-point scale (0=never/not true; 1=sometimes/somewhat true; 2=often/very true). Endorsing one or more item as “often/very true” suggests that the child perceives the event as potentially traumatic and qualifies the child to participate in the study. The screen is derived from the Acute Stress Checklist for Children.

### Intervention use, satisfaction, and engagement

#### Online tracking

Automated electronic tracking during participants’ use of the web-based intervention will capture the time and date of each log-in, duration of each session in which the participant uses Coping Coach, completion of each intervention task or module, and participant responses to questions and activities that are built into the interactive intervention.

#### The satisfaction and engagement questionnaire (parallel parent and child versions)

This questionnaire was created for this study and is designed to gather overall impressions of and satisfaction with the Coping Coach intervention. It is divided into three sections: (1) several open-ended questions elicit strengths and areas for improvement, (2) items rated yes/no and on a 3-point Likert scale (yes, maybe, and no) ask the respondent to assess the intervention's visual appeal, functionality, and the trustworthiness and comprehensibility of the intervention content, and (3) several open-ended questions assess how families engaged in the intervention at home and any barriers incurred to completing the Coping Coach activities at home.

### Trauma history

#### The trauma screen fromUCLA PTSD index for DSM-IV

This trauma history measure is comprised of 12 items that assess prior exposure to a variety of traumatic events (e.g., natural disaster, accident, war, violence, and medical treatment) and is intended for use with children aged 7 and older. (Pynoos, Rodriguez, Steinberg, Stuber, & Frederick, 1998).

### Coping and coping assistance

#### The How I Coped Under Pressure Scale

The How I Coped Under Pressure Scale (HICUPS) is a self-report questionnaire that is used to assess children's use of adaptive coping strategies with regard to their recent medical event (Ayers, Sandler, West, & Roosa, 1996). The HICUPS has well-established reliability and validity. The measure has been used with children of different ethnicities and socioeconomic status facing a variety of stressors (e.g., Landolt, Vollrath, & Ribi, 2002; Lengua, Long, & Meltzoff, 2006). In this study, specific subscales that match content covered in the intervention will be administered (Positive Cognitive Restructuring, Distraction, Support Seeking, and Avoidance Coping).

#### The Parent Socialization of Coping Questionnaire

The Parent Socialization of Coping Questionnaire (PSCQ) parallels the HICUPS and assesses parent encouragement or coaching of children's coping strategies (Miller, Kliewer, Hepworth, & Sandler, 1994). Parents rate the extent to which they have encouraged or discouraged each specific child coping strategy by responding on a 7-point Likert scale. Parallel PSCQ subscales will be administered to parents. Research has suggested that the PSCQ is a reliable and valid assessment of parental coping assistance.

### Cognitions and appraisals

#### The Child Posttraumatic Cognitions Inventory

The Child Posttraumatic Cognitions Inventory (CPTCI) is a 25-item scale adapted from the Posttraumatic Cognitions Inventory [developed for adults (Foa, Ehlers, Clark, Tolin, & Orsillo, 1999)] to be used with children (Meiser-Stedman, Smith, et al., 2009). The CPTCI was developed and validated within a large sample of children and adolescents aged 6–18 years. Principal components analysis suggested a two-component structure, labeled “permanent and disturbing change” and “fragile person in a scary world”. Each subscale has good internal consistency, test–retest reliability, convergent validity, and discriminant validity. The reliability and validity of these sub-scales was confirmed both in the acute phase and several months after a trauma.

#### The Adaptive Appraisals Questionnaire.

The Adaptive Appraisals Questionnaire (AAQ) is a 21-item measure that assesses the extent to which a child perceives the medical event as time limited or in the past, expects a successful outcome, sees potential benefit, and perceives personal strength or self-efficacy regarding the medical event (Ellis, 2008).

### Health-related quality of life

#### The Pediatric Quality of Life Inventory

The Pediatric Quality of Life Inventory (PedsQL) is a well-validated measure of child HRQOL (Varni, Seid, & Rode, 1999). It is developmentally appropriate, with child self-report and parent-report instruments available for children aged 2–18 years. The PedsQL has four scales with a total of 23 items: Physical health/physical functioning (eight items), Psychosocial health/emotional functioning (five items), Psychosocial health/social functioning (five items), and Psychosocial health/school functioning (five items).

### Posttraumatic stress symptom

#### The Child PTSD Symptom Scale

The Child PTSD Symptom Scale (CPSS) is a 24-item self-report instrument that yields both a PTSD symptom severity score (possible range 0–51) and a determination of likely PTSD diagnostic status (Foa, Johnson, Feeny, & Treadwell, 2001). Seventeen CPSS items correspond to the DSM-IV symptom criteria and seven items assess impairment from those symptoms. The CPSS has shown excellent internal consistency (*α*=0.89), test–retest reliability (0.84), and convergent validity with structured clinical interview measures of PTSD (Foa et al., 2001). Confirmatory factor analyses also support the construct validity of the measure (Kassam-Adams, Marsac, & Cirilli, 2010).

##### The PTSD Checklist

The PTSD Checklist (PCL) is a well-validated 17-item self-report questionnaire that yields both a PTSD symptom severity score (possible range 17–85) and a determination of likely PTSD diagnostic status (Blanchard, Jones-Alexander, Buckley, & Forneris, 1996; Weathers & Ford, 1996). PCL items correspond to DSM-IV symptom criteria. The PCL has demonstrated strong internal consistency (*α*=0.94), test–retest reliability, and convergent and discriminant validity. The PCL has been utilized (Manne, Du Hamel, Gallelli, Sorgen, & Redd, 1998) to assess PTSD symptoms in parents of ill or injured children.

### Child health and recovery

#### The health and recovery questionnaire

This 8-item questionnaire will be used to collect information at the 12-week follow-up assessment about help-seeking and services used (health care, mental health care, informal psychosocial support) over a specified period after an index medical event (Marsac, Cirilli, Kassam-Adams, & Winston, 2011).

### Sample size

A primary goal of this RCT is to estimate effect sizes for a later full-scale RCT. Within the constraints of a pilot study, there will be reasonable power to detect a clinically meaningful effect for proximal outcomes (appraisals and coping at 6 weeks) and child health outcomes (PTSS and HRQOL at 12 and 18 weeks). With a sample of 70 participants (35 randomized to each condition), we project that we will have 60 (85%) retained to all follow-up assessments. With this sample size, a difference of 0.5 SD between conditions [based on an Analysis of covariance (ANCOVA)] with 80% power, while controlling for *α* =0.05, can be detected.

### Data analysis

ANCOVA is the primary analytic approach for examining outcomes and for initial estimation of effect sizes. ANCOVA can adjust for baseline differences between groups (intervention vs. waitlist control), as imbalances may occur despite randomization. The dependent variable in each ANCOVA will be a 6-week proximal outcome or a 12- or 18-week child health outcome, the corresponding baseline score of the outcome measure will be the covariate, and group (condition) will be the qualitative factor. Other covariates that may help explain variation in intervention effects (e.g., age, gender, prior trauma, and parent PTSS) will be considered for inclusion in these analyses. Multivariable regression analysis will also be used to examine the hypothesized role of proximal outcomes in predicting each health outcome. An intent-to-treat approach will be applied to help handle missing data.

## Discussion

The high prevalence and significant impact of acute traumas on children's functioning warrant innovative approaches of delivering effective secondary prevention of psychosocial sequelae. Coping Coach translates research on etiology and malleable risk and protective factors into a novel web-based intervention. The intervention integrates evidence-based components of CBT techniques and Internet interventions, applying these to prevention by targeting early appraisals, coping, and support-seeking. Coping Coach is designed to require interaction with the game content to facilitate engagement and learning. The current RCT will provide us with pilot data to prepare for a larger-scale evaluation of Coping Coach. Specifically, this study will examine the feasibility of the Coping Coach intervention as well as the study procedures. In addition, the current RCT represents a unique opportunity to estimate the efficacy of Coping Coach in promoting positive child health outcomes and preventing or reducing negative psychological sequelae. Toward this goal we will examine proximal outcomes of appraisals and coping along with long-term child health outcomes, PTSS and HRQOL. We expect that the results of this RCT will augment the existing literature by producing new information (pilot data) on the effectiveness of web-based preventive interventions for children after acute medical trauma.

Among the strengths of this study is its incorporation of assessments with both children and parents to evaluate clinically meaningful short- and long-term effects of Coping Coach. Data from this RCT will help to refine our program theory and potentially modify existing conceptual models for the development of psychological symptoms after an acute medical event (i.e., hypothesized etiological mechanisms). This RCT will also enable us to estimate effect sizes for proximal and child health outcomes and inform modifications of Coping Coach prior to a larger scale trial. The electronic tracking built into the study design allows us to examine actual completion of the intervention modules to determine if there is a necessary minimum intervention “dose” and allows us to evaluate how children interact with game content. Finally, this RCT will allow us to evaluate factors that may impact child engagement and adherence and to identify ways to increase potency of intervention effects.

Possible limitations of this RCT include technical difficulties that may be experienced by participants. To meet this challenge, we have worked to ensure accessibility and functionality of the intervention. Another potential limitation is that some participants may not finish all intervention modules after discharge from the hospital. We will attempt to minimize this by sending tailored, weekly reminders to children and families and providing a small incentive for children who complete all Coping Coach modules. In addition, we will track the completion of the intervention electronically, so that we will be able to determine what intervention components were completed by each participant. Other potential limitations include recruitment challenges or possible sampling bias. We will attempt to prevent this by closely monitoring weekly recruitment and retention rates. We will adjust strategies as needed to achieve recruitment targets, and address any significant deviations of sample demographics from the pool of eligible patients.

If results suggest that Coping Coach can prevent emotional sequelae and improve child health outcomes, the intervention would provide a promising avenue to help children cope in the aftermath of a traumatic medical event. Given its web-based modality, Coping Coach can provide widely accessible tools to promote positive child health outcomes. In addition, Coping Coach could be provided as a resource that physicians, mental health professionals, social workers, and teachers could recommend for children at risk for PTSS.
